# Advancements in Immune Checkpoint-Based Immunotherapy for Triple-Negative Breast Cancer

**DOI:** 10.3390/cimb48060615

**Published:** 2026-06-12

**Authors:** Dexian Wei, Yuan Zhang, Yanlin Wu, Liqun Ren, Qing He

**Affiliations:** 1 State Key Laboratory of Drug Regulatory Sciences, National Institutes for Food and Drug Control, Beijing 102629, China; weidx24@mails.jlu.edu.cn (D.W.); zhyuan_cn@126.com (Y.Z.); wyljoe@126.com (Y.W.); 2 Department of Experimental Pharmacology and Toxicology, School of Pharmaceutical Sciences, Jilin University, Changchun 130021, China

**Keywords:** triple-negative breast cancer, immunotherapy, immune checkpoints, immune checkpoint blockade

## Abstract

Triple-negative breast cancer (TNBC), characterized by the lack of estrogen receptor (ER), progesterone receptor (PR), and human epidermal growth factor receptor 2 (HER2) expression, is a highly aggressive molecular subtype with high recurrence and metastasis rates. Due to the absence of reliable molecular targets, surgery combined with chemotherapy remains the mainstay of clinical treatment. In recent years, immunotherapy has provided new strategies for TNBC management. Immune checkpoints are key regulatory molecules that maintain immune homeostasis, and blocking these checkpoints can restore T cell activity and enhance tumor cell killing. Immune checkpoint inhibitors (ICIs) have demonstrated clinical benefit, particularly in combination with chemotherapy for patients with locally advanced or metastatic TNBC. This review focuses on immune checkpoint–based immunotherapy in TNBC, providing an overview from mechanistic insights to clinical applications and emerging therapeutic strategies. In addition to ICIs, we discuss alternative approaches, such as bispecific antibodies, antibody–drug conjugates (ADCs), chimeric antigen receptor T cell (CAR-T) therapy, tumor vaccines, and oncolytic viruses (OVs), highlighting their current research progress and clinical applications in TNBC treatment.

## 1. Introduction to Immunotherapy in TNBC

Breast cancer is a malignant tumor that remains a major global public health concern and poses one of the leading threats to women’s health worldwide [1,2]. Compared with other cancer types, breast cancer accounts for a greater loss of disability-adjusted life years (DALYs) among women worldwide. By 2050, its global incidence is projected to increase by 38%, and annual breast cancer related deaths are expected to more than double, reaching approximately 1.1 million [3,4,5]. These studies underscore the urgent need to develop more effective therapeutic strategies for patients with breast cancer. Among the different molecular subtypes of breast cancer, triple-negative breast cancer (TNBC) is characterized by the lack of expression of estrogen receptor (ER), progesterone receptor (PR), and human epidermal growth factor receptor 2 (HER2), accounting for approximately 8–13% of all breast cancer cases [6]. Meanwhile, TNBC is also the most aggressive subtype, exhibiting the highest rates of postoperative recurrence and mortality, and is associated with poor overall survival (OS). Currently, chemotherapy combined with surgery remains the most effective treatment strategy for TNBC. Owing to the absence of ER, PR and HER2 receptors, TNBC lacks molecular targets in clinical practice, making targeted therapy more challenging than in other breast cancer subtypes [7]. Conventional treatments, including surgery, radiotherapy, and chemotherapy, remain limited in efficacy, and the effective therapeutic options have long been lacking [8].

In recent years, cancer immunotherapy has made remarkable progress and has emerged as an important therapeutic option for cancer. Approaches include immune checkpoint inhibitors (ICIs), chimeric antigen receptor T cell (CAR-T) therapy, cancer vaccines, and oncolytic viruses (OVs) [9]. The fundamental principle of cancer immunotherapy is to activate and enhance the host immune system, thereby enabling it to recognize and eliminate tumor cells and modulate the tumor microenvironment (TME). With rapid advances in tumor biology, immunology, and molecular biology, investigations into the TME, an essential component influencing the efficacy of cancer immunotherapy, have become increasingly comprehensive and in-depth. The TME consists of tumor cells, immune cells, fibroblasts, endothelial cells, extracellular matrix components, and various signaling molecules, including cytokines and chemokines [10,11]. Studies have shown that the cellular components of the TME, particularly immune cells such as T cells, natural killer (NK) cells, dendritic cells (DCs), macrophages, and regulatory T cells (Tregs), play crucial roles in regulating tumor progression, metastasis, and responses to therapy [12].

Immune checkpoints, such as programmed cell death protein 1 (PD-1), programmed cell death ligand-1 (PD-L1), are regulatory molecules of that play a critical role in maintaining immune homeostasis by modulating the activation and inhibition of immune responses [13,14]. Tumor cells can exploit these checkpoint pathways to escape from immunosurveillance by suppressing antitumor immune responses within the TME. Consequently, immune checkpoint blockade (ICB) therapy has achieved remarkable progress in cancer treatment. By blocking inhibitory signaling pathways, checkpoint inhibitors can reprogram the immunosuppressive TME, restore T cell activity, enhance antitumor cytotoxicity, and thereby suppress tumor progression [15,16].

Although most breast cancer subtypes are generally considered immunologically “cold” tumors characterized by low T cell infiltration and low tumor mutation burden (TMB), TNBC exhibits a relatively more immunogenic TME. Compared with other breast cancer subtypes, TNBC is associated with increased tumor infiltrating lymphocytes (TIL) infiltration, elevated PD-L1 expression, and higher genomic instability, contributing to its distinct immunological characteristics. Notably, strong interactions exist between immune checkpoint pathways and various cellular components of the TME. ICB has been shown to modulate or reprogram intercellular interactions within the TNBC TME, thereby reshaping antitumor immune responses [17]. Investigations have shown that DCs upregulate PD-L1 expression upon antigen uptake, which may protect them from cytotoxicity mediated by activated T cells. In addition, TNBC exhibits upregulated expression of PD-L1. Therefore, assessment of PD-L1 expression may help identify patients who are more likely to benefit from ICB, as higher PD-L1 levels have been associated with improved responses to PD-1/PD-L1-targeted therapies in certain clinical settings. Collectively, these findings suggest that TNBC is a promising candidate for immunotherapeutic strategies [18,19,20]. However, substantial intertumoral heterogeneity means that many TNBC tumors still exhibit immunologically “cold” phenotypes, thereby limiting the efficacy of ICIs. Consequently, emerging immunotherapeutic strategies aimed at converting “cold” tumors into immunologically active “hot” tumors by enhancing tumor immunogenicity and promoting T cell infiltration have become promising approaches for improving the therapeutic efficacy of TNBC immunotherapy [21].

**Figure 1:**
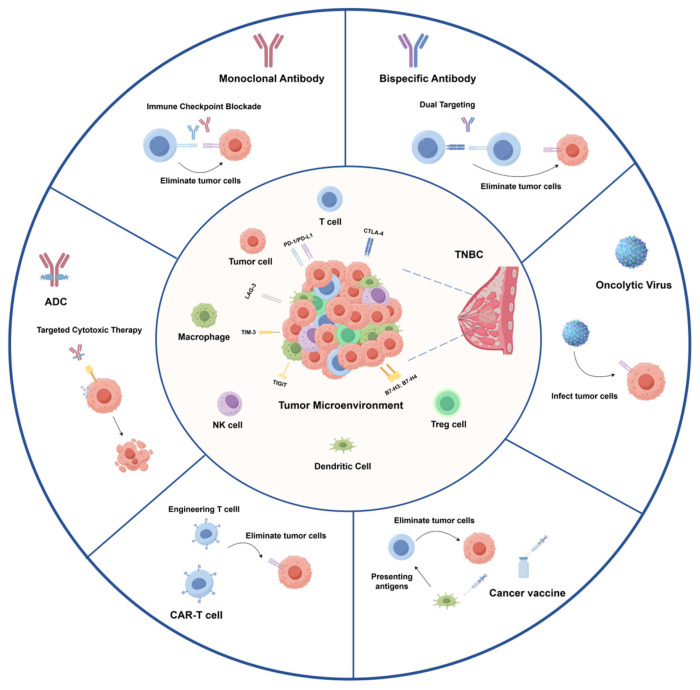
Diagram of various immunotherapies targeted immune checkpoints in the TNBC tumor microenvironment. This figure was drawn by Figdraw. TNBC—Triple-Negative Breast Cancer; ADC—Antibody–drug conjugate; NK cell—Natural killer cell.

Currently, several immune checkpoint clinical trials have demonstrated the potential benefits of immunotherapy for TNBC patients. In particular, ICIs combined with chemotherapy have demonstrated significant clinical benefit and have been approved for the treatment of PD-L1-positive locally advanced or metastatic TNBC [22,23]. However, major challenges still remain, particularly the lack of predictive biomarkers for identifying patients who may benefit from ICIs. Herein, this review focuses on immune checkpoint–based immunotherapies, and provides a concise summary of immune checkpoint pathways, recent advances and clinical trials in ICIs, predictive biomarkers associated with ICI response, as well as emerging strategies aimed at enhancing immune checkpoint-targeted immunotherapy, including antibody–drug conjugates (ADCs), CAR-T cells, cancer vaccines, and OVs in the treatment of TNBC. (Immune checkpoints in the TME of TNBC targeted by various immunotherapies are shown in Figure 1).

## 2. Mechanistic Insights and Clinical Applications of ICIs Targeting PD-1/PD-L1 in TNBC

### 2.1. PD-1/PD-L1

Programmed death 1 (PD-1) and its ligands PD-L1 are key members of immune checkpoint pathways, in which PD-1 is a member of the CD28 family, while PD-L1 is a member of the B7 family. PD-1, also known as CD279, is a key immunosuppressive molecule primarily expressed on immune cells, including T cells, B cells, NK cells, and myeloid cells. Structurally, PD-1 contains two cytoplasmic motifs: the immunoreceptor tyrosine-based inhibitory motif (ITIM) and the immunoreceptor tyrosine-based switch motif (ITSM) [24]. PD-L1, also known as B7-H1 or CD274, is a type I transmembrane protein primarily expressed on T and B lymphocytes as well as APCs. It functions as a key inhibitory immune checkpoint involved in the regulation of antitumor immunotherapy [25]. During tumor progression, recognition of tumor-associated antigens (TAAs) presented by major histocompatibility complex (MHC) molecules on tumor cells by T cells triggers the production of proinflammatory cytokines, including tumor necrosis factor-α (TNF-α) and interferon-γ (IFN-γ). These cytokines, particularly IFN-γ, can subsequently induce the upregulation of PD-L1 expression on tumor cells and other cells within the TME. In parallel, antigen stimulation activates multiple transcription factors in T cells, such as nuclear factor of activated T cells c1 (NFATc1) and activator protein-1 (AP-1), which promote the transcriptional upregulation of PD-1. Sustained activation of the PD-1/PD-L1 axis ultimately contributes to T cell functional exhaustion and the establishment of immune tolerance within the TME [5,26]. The interaction between PD-1 and PD-L1 suppresses the transcription and translation of genes and cytokines essential for T cell activation, thereby enabling tumor cells to evade immune surveillance and cytotoxicity, ultimately leading to immune escape [27].

Studies have shown that upon binding to its ligand PD-L1, PD-1 undergoes phosphorylation of its cytoplasmic ITIM and ITSMs, primarily mediated by the Src family kinase Lck. This event leads to the subsequent recruitment of Src homology region 2 domain–containing phosphatases, predominantly SHP-2, which dephosphorylate key signaling components of the T cell receptor (TCR) and CD28 co-stimulatory pathways, including CD3ζ and ZAP70. Engagement of PD-1 by PD-L1 further suppresses downstream signaling cascades such as the PI3K–Akt–mTOR and Ras–MEK–ERK pathways. Consequently, PD-1/PD-L1 interaction disrupts TCR signaling and inhibits T cell activation [5,28,29].

Beyond directly inhibiting T cell signaling, the PD-1/PD-L1 axis can also exert immunosuppressive effects through other cellular components within the TME. In DCs, PD-L1 can bind to CD80 in cis, thereby disrupting the CD80–CD28 co-stimulatory interaction that is required for effective T cell activation, ultimately suppressing antitumor immune responses [30]. Conversely, blockade of PD-L1 on DCs has been shown to significantly enhance CD8^+^ T cell proliferation and granzyme B production, thereby restoring T cell effector function and cytotoxic activity [31]. In addition, interactions between Tumor-Associated Macrophages (TAMs) and TNBC cells can influence the therapeutic efficacy of PD-1/PD-L1 inhibitors. Recent studies have shown that inhibition of Notch signaling or blockade of cytokines such as CCL2 and IL-1β, which mediate TAM recruitment, can reverse the immunosuppressive microenvironment of TNBC and enhance the therapeutic efficacy of ICB [32]. In addition, blocking IL1R2 has been shown to reduce macrophage recruitment and TAM polarization, suppress TNBC cell growth, and upregulate PD-L1 expression, thereby enhancing the response to anti-PD-1 therapy in TNBC [33].

### 2.2. Research and Clinical Applications of ICIs Targeting PD-1/PD-L1 in TNBC

ICIs targeting the PD-1/PD-L1 axis have been approved by the U.S. Food and Drug Administration (FDA) for the treatment of TNBC patients in specific clinical settings, particularly in combination with chemotherapy. Blockade of the PD-1/PD-L1 pathway has emerged as one of the most extensively studied and clinically validated immune checkpoint inhibition strategies [34,35,36]. ICIs function by blocking the interaction between checkpoint proteins and their ligands, thereby preventing inhibitory signal transduction, restoring T cell activation, and enabling T cells to effectively eliminate tumor cells expressing immune checkpoint [37,38]. Notably, TNBC has the characteristics of strong immune infiltration, high mutation load and genomic instability, and is more likely to produce new antigens than other subtypes, making it more suitable for immunotherapy. In addition, studies have shown that TNBC has high expression of immune checkpoint molecules, further supporting its suitability for anti-PD-1/PD-L1 immune checkpoint blockade–based therapies [24,39,40].

#### 2.2.1. ICIs Targeting PD-1/PD-L1

PD-1 and PD-L1 are currently recognized as the most effective immune checkpoint targets in TNBC. In 2019, the FDA approved atezolizumab in combination with nab-paclitaxel for the treatment of patients with locally advanced or metastatic TNBC. Subsequently, in 2020, the FDA approved pembrolizumab combined with chemotherapy for patients with locally recurrent, unresectable, or metastatic PD-L1-positive TNBC [41].

The phase I KEYNOTE-012 study evaluated the safety and antitumor activity of anti-PD-1 antibody pembrolizumab in TNBC patients. The results demonstrated that most treatment-related adverse events were mild, with an overall response rate (ORR) of 18.5% and a median time to response of 17.9 weeks, thereby providing preliminary evidence of the clinical activity and safety profile of pembrolizumab in TNBC [42]. Subsequently, in the phase II KEYNOTE-086 study, pembrolizumab demonstrated a manageable clinical safety profile in TNBC patients, with low rates of adverse events. Notably, it exhibited more durable antitumor activity in PD-L1-positive metastatic TNBC (mTNBC) patients, achieving an ORR of 21.4% [43,44]. The phase III KEYNOTE-522 trial demonstrated that the combination of pembrolizumab and neoadjuvant chemotherapy significantly improved overall survival compared with neoadjuvant chemotherapy alone in patients with early-stage TNBC. Furthermore, the phase III KEYNOTE-355 study demonstrated that pembrolizumab, as monotherapy or in combination with chemotherapy, significantly prolonged median overall survival (mOS) compared with chemotherapy alone in patients with advanced TNBC whose tumors were PD-L1 positive with a combined positive score (CPS) ≥ 10 [45,46].

Regarding the previously approved anti-PD-L1 inhibitor atezolizumab, the IMpassion130 trial demonstrated that atezolizumab combined with nab-paclitaxel significantly improved median progression-free survival (mPFS) in patients with PD-L1-positive metastatic TNBC. In addition, the neoadjuvant IMpassion031 study showed that the addition of atezolizumab to chemotherapy significantly increased the pathological complete response (pCR) rate in patients with early-stage TNBC while maintaining a manageable safety profile [47,48,49]. However, subsequent clinical trials yielded less favorable outcomes. The IMpassion131 study demonstrated that the combination of atezolizumab and paclitaxel did not improve PFS or OS compared with paclitaxel alone in TNBC patients. Moreover, results from the IMpassion132 trial indicated that atezolizumab was associated with poor prognosis in TNBC patients who relapsed within 12 months, and its combination with chemotherapy failed to significantly improve OS [50,51]. These unsuccessful trials have undoubtedly impacted the continued approval of atezolizumab for TNBC indications. In 2021, the FDA withdrew the indication for atezolizumab in combination with nab-paclitaxel for PD-L1-positive metastatic TNBC following inconsistent clinical outcomes across the IMpassion trials.

Clinical trials of anti-PD-1/PD-L1 monoclonal antibodies pembrolizumab and atezolizumab in TNBC are shown in Table 1. Taken together, these clinical trials demonstrate that anti-PD-1 antibody pembrolizumab combined with chemotherapy, has achieved meaningful clinical benefit in patients with PD-L1-positive mTNBC, highlighting the substantial progress of ICB in TNBC treatment. In contrast, clinical studies evaluating the anti-PD-L1 antibody atezolizumab have yielded inconsistent results across different trials, potentially reflecting differences in patient selection, prior exposure to chemotherapy, and the substantial heterogeneity of TNBC. These findings indicate that, although ICIs have significantly expanded therapeutic options for TNBC, major challenges remain, including limited response rates, primary and acquired resistance, and the need for more reliable predictive biomarkers. All the clinical trials of immunotherapy for TNBC registered at clinicaltrials.gov.

In addition to the two ICIs mentioned above, multiple novel ICIs are currently under development or have started clinical trials for TNBC. Durvalumab, a PD-L1 inhibitor that has demonstrated efficacy in bladder cancer and small cell lung cancer, has also shown promise in mTNBC. Clinical trials have reported that Durvalumab in combination with chemotherapy can increase the pCR rate in TNBC patients [52]. Nivolumab is a humanized IgG4 monoclonal antibody targeting PD-1. It has been approved for the treatment of non-small cell lung cancer and unresectable or metastatic melanoma. In TNBC, studies analyzing serum analyte levels in patients with residual disease after neoadjuvant chemotherapy have shown that treatment with nivolumab—either as monotherapy or in combination with chemotherapy—enhances immune activation compared with chemotherapy alone [53,54]. Avelumab, an IgG1 anti-PD-L1 ICI approved for the treatment of patients with advanced urothelial carcinoma, can induce antibody-dependent cell-mediated cytotoxicity against tumor cells [55]. In clinical studies, avelumab in combination with PARP inhibitors was found to prolong the duration of response (DOR) in TNBC patients, highlighting the potential of combining ICIs with PARP inhibition [56]. At the same time, studies have shown that avelumab does not significantly improve DFS in high-risk early-stage TNBC patients, but it does reduce the risk of death [57].

Clinical trials investigating other anti-PD-1/PD-L1 monoclonal antibodies in TNBC are summarized in Table 2. Compared with the clinically established antibodies pembrolizumab and atezolizumab, several emerging PD-1/PD-L1-targeting antibodies have incorporated structural modifications, particularly the IgG backbone, aiming to improve antitumor activity and safety profiles. Early-phase clinical studies have demonstrated encouraging preliminary efficacy in selected TNBC populations. However, unlike the mature phase III clinical evidence supporting pembrolizumab- and atezolizumab-based regimens, most of these novel antibodies remain in phase I/II clinical development, with many studies still ongoing or actively recruiting. Therefore, further large-scale clinical validation is required to determine their long-term therapeutic value and clinical applicability in TNBC.

#### 2.2.2. Combination Therapies with ICIs Targeting PD-1/PD-L1

In recent years, the efficacy of PD-1/PD-L1 blockade as monotherapy has remained limited, with minimal impact on clinical outcomes in patients with advanced TNBC, thereby necessitating combination with other therapeutic modalities. Among these strategies, the combination of chemotherapy with PD-1/PD-L1 inhibitors has demonstrated improved clinical outcomes in TNBC compared with immunotherapy alone. Currently, combining ICIs with chemotherapy is the primary approach for anti-tumor immunotherapy. Chemotherapy, as a first-line treatment used in TNBC, can enhance antitumor immunity by inducing tumor cell apoptosis and facilitating CD8^+^ T cell infiltration. In addition, studies have shown that chemotherapy drugs can induce immunogenic cell death (ICD) in tumor cells. Dying tumor cells release TAAs and damage associated molecular patterns (DAMPs), which stimulate DC maturation and trigger antigen-specific immune responses [58]. Several clinical trials have demonstrated that, compared with neoadjuvant chemotherapy alone, PD-1/PD-L1 inhibitors such as pembrolizumab and durvalumab combined with chemotherapeutic agents—including paclitaxel, anthracyclines, and platinum compounds—can significantly improve the pCR rate and overall survival in TNBC patients. Collectively, these immunomodulatory effects help convert an immunologically “cold” TME into a more immunogenic state, thereby potentiating the efficacy of immune checkpoint blockade [59,60,61,62,63].

The combination of ICIs with other immunotherapies is also an active area of investigation. For instance, ADCs, which have emerged as a promising therapeutic strategy in TNBC. ADCs targeting Trop-2, such as Sacituzumab govitecan (SG), in combination with pembrolizumab or atezolizumab, have been tested, with phase II clinical trials currently underway in TNBC patients. Another new Trop-2 targeting ADC, Dato-DXd, in combination with Durvalumab, has shown potential as a therapeutic strategy for TNBC in clinical studies [64,65]. In addition, combining ICIs with novel immunotherapies shows great potential. For example, several personalized neoantigen tumor vaccines plus ICI have entered phase I clinical trials in TNBC patients [66]; in addition, novel antisense oligonucleotide AZD8701, targeting FoxP3 in Tregs in combination with Durvalumab, has demonstrated clinical feasibility in patients with advanced solid tumors, including TNBC [67].

Meanwhile, ICIs can also be combined with targeted inhibitors for the treatment of TNBC, among which PARP inhibitors are the most representative. Research have shown that PARP inhibition can upregulate PD-L1 expression, providing a rationale for combining PARP inhibitors with PD-1 blockade. In the KEYNOTE-162 trial, niraparib, a PARP1/PARP2 inhibitor, in combination with pembrolizumab, demonstrated anti-tumor activity in patients with advanced or metastatic TNBC, with a manageable safety profile. Similarly, olaparib, a PARP inhibitor used in BRCA-mutated breast cancer, combined with pembrolizumab as adjuvant therapy, has shown clinical benefit in high-risk, early-stage, HER2-negative, BRCA1/2-mutated breast cancer [68,69,70].

Clinical trials investigating novel anti-PD-1/PD-L1-based combination strategies in TNBC are summarized in Table 3. In recent years, ICIs have been extensively combined with ADCs, PARP inhibitors, targeted agents, cancer vaccines, and other therapeutic modalities in an effort to enhance tumor immunogenicity, promote T cell infiltration, and overcome resistance to immune checkpoint blockade. Several combination regimens have already progressed to phase II and phase III clinical trials, reflecting the growing translational potential of combination immunotherapy in TNBC. Although many ongoing studies remain in the recruitment or active evaluation stage, preliminary clinical evidence suggests that selected combination strategies may further improve therapeutic efficacy compared with ICI monotherapy.

### 2.3. Immune-Related Adverse Events (irAEs) of ICIs

ICIs, whether administered as monotherapy or in combination with chemotherapy and other agents, are associated with immune-related adverse events (irAEs) resulting from nonspecific immune activation, ranging from mild, asymptomatic findings (Grade 1) to life-threatening conditions requiring urgent intervention (Grade 4) and even fatal outcomes (Grade 5). These toxicities most commonly affect the skin, gastrointestinal tract, liver, and endocrine organs, such as thyroid dysfunction [71,72,73]. Across the clinical trials summarized in TNBC, most irAEs are mild to moderate (Grade 1–2). However, grade ≥ 3 immune-related and treatment-associated adverse events were reported in a subset of patients and often required treatment interruption or discontinuation. Following clinical recovery, therapy can typically be resumed with appropriate dose modification [49,54,56,61,74,75].

In addition, combination regimens, particularly those including chemotherapy, ADCs, PARP inhibitors, or other immune checkpoint–targeted agents, are generally associated with increased frequencies of treatment-related adverse events and overlapping toxicities compared with ICI monotherapy. Adverse events associated with combination regimens include treatment-related toxicities such as anemia, pyrexia, and febrile neutropenia, as well as potentially severe immune-mediated toxicities such as colitis, hepatitis, myocarditis, and pneumonitis [76]. Several clinical trials have reported increased incidences of grade ≥ 3 adverse events in patients receiving ICI-based combination therapies. Management strategies include careful monitoring, dose modification, temporary treatment interruption, and corticosteroid-based immunosuppressive therapy for severe irAEs [77]. Therefore, the development of emerging combination strategies should carefully balance antitumor efficacy with treatment-related toxicity, particularly in comparison with currently established pembrolizumab-based regimens in TNBC.

Notably, the risk–benefit balance may differ substantially between early-stage and mTNBC. In early-stage TNBC, treatment strategies are generally administered with curative intent, and patients may tolerate higher toxicity burdens to maximize long-term survival benefit. Nevertheless, careful patient selection remains critical to avoid overtreatment while pursuing cure. In contrast, in mTNBC, intensified combination regimens aimed at improving immunotherapeutic efficacy may increase treatment discontinuation, cumulative toxicity burden, and negatively impact quality of life, particularly in heavily pretreated patients with limited therapeutic tolerance. Strategies to optimize the risk–benefit profile in this setting may include the use of predictive biomarkers, individualized dose adjustments, and vigilant monitoring to balance efficacy and safety [78].

Although several ICIs are currently under development or in clinical evaluation and have demonstrated potential efficacy, a major challenge for their future clinical application is maintaining therapeutic effectiveness while improving safety. Therefore, it is essential to develop rational combination strategies that maximize antitumor efficacy while minimizing additional toxicity.

### 2.4. Predictive Biomarkers for ICIs in TNBC

Compared with other molecular subtypes of breast cancer, TNBC exhibits relatively higher immunogenicity, characterized by elevated PD-L1 expression, increased TILs, and a higher somatic mutation rate. These immunological features have been extensively investigated as potential biomarkers for predicting responses to ICIs in TNBC and for identifying patients most likely to benefit from immunotherapy [79,80].

Despite this immunogenic potential, resistance to ICIs remains a major challenge in TNBC. Resistance can be broadly classified into primary resistance, where patients fail to achieve an initial clinical response, and acquired resistance, where patients initially respond but subsequently relapse after a period of disease control. Understanding and monitoring this resistance patterns highlight the complex and dynamic interactions between tumors and the immune system and underscore the critical role of predictive biomarkers not only for patient stratification but also for potentially overcoming ICI resistance.

#### 2.4.1. PD-L1 Expression

Assessment of PD-L1 expression in TNBC has important clinical implications for patient selection and therapeutic decision-making, as higher PD-L1 expression, commonly defined as a combined positive score (CPS) ≥ 10, has been associated with improved responses to ICIs. Currently, PD-L1 expression evaluated by CPS represents the most clinically established biomarker for predicting responses to anti-PD-1/PD-L1 therapies, particularly in locally advanced and metastatic TNBC. PD-L1 CPS has been widely used as a stratification biomarker in major clinical trials evaluating pembrolizumab and has demonstrated predictive value for pCR and clinical benefit in selected TNBC populations [37,81].

However, PD-L1 expression exhibits substantial spatial and temporal heterogeneity within the TME. Its expression may dynamically evolve during disease progression and vary across different metastatic sites. In addition, the predictive accuracy of PD-L1 CPS remains limited, and significant variability exists among different PD-L1 detection assays and scoring systems. Therefore, PD-L1 expression alone is insufficient to accurately predict which patients will derive durable clinical benefit from ICIs [82].

#### 2.4.2. TILs

TILs, including CD8^+^ cytotoxic T cells, CD4^+^ helper T cells, and B cells, play critical roles in antitumor immunity and immunotherapy in TNBC. Tumor antigens can recruit and activate TILs to secrete cytokines such as IFN-γ, which promote tumor-cell killing and subsequently upregulate PD-L1 expression within the TME. TILs have therefore been extensively investigated as potential biomarkers for immunotherapy response in TNBC. Several studies have demonstrated that higher densities of TILs, particularly CD8^+^ cytotoxic T cells, are significantly associated with improved responses to neoadjuvant chemotherapy and increased pCR rates in TNBC patients [25,83].

#### 2.4.3. TMB

TMB is another important biomarker that has been investigated in cancer immunotherapy and is generally defined as the total number of somatic mutations per megabase of genomic sequence, including base substitutions, insertions, and deletions. Higher TMB levels, commonly defined as >10 mutations/Mb, may promote the generation of neoantigens, thereby enhancing antigen presentation by APCs and facilitating T cell recognition and activation. Compared with other breast cancer subtypes, TNBC generally exhibits a relatively higher somatic mutation rate, suggesting greater immunotherapeutic potential. Several studies have indicated that elevated TMB may correlate with improved responses to ICIs in certain TNBC patients [84,85].

#### 2.4.4. HLA Expression

Human leukocyte antigen (HLA) expression is closely associated with antigen presentation and T cell–mediated antitumor immunity. TNBC exhibits relatively elevated expression of several HLA-related genes compared with other breast cancer subtypes, potentially contributing to enhanced neoantigen presentation and immune recognition [86]. These characteristics may partially explain the relatively higher immunogenicity observed in TNBC and may influence the therapeutic responsiveness to ICIs.

Collectively, while PD-L1 expression, TILs, TMB, and HLA-related features provide valuable predictive information, no single biomarker is sufficient to reliably predict ICI efficacy due to the molecular and immunological heterogeneity of TNBC. Integrated biomarker strategies, potentially combining multiple immune and genomic parameters, remain a crucial focus for improving patient selection, enhancing the clinical efficacy and overcoming resistance mechanisms in TNBC immunotherapy.

## 3. Mechanistic Insights and Clinical Applications of ICIs Targeting Alternative Immune Checkpoints in TNBC

Despite the clinical success of ICIs targeting the PD-1/PD-L1 axis, their clinical application remains associated with several important limitations. These include primary and acquired immune tolerance to PD-1/PD-L1 blockade, as well as the persistent challenge of balancing therapeutic efficacy with irAEs. Consequently, there remains an increasing recognition that reliance on the PD-1/PD-L1 axis alone is insufficient for immunotherapy in TNBC, underscoring the urgent need for additional predictive biomarkers and alternative immunomodulatory targets. Recently, a growing number of immune checkpoints beyond PD-1 and PD-L1—such as CTLA-4, LAG-3, TIM-3, TIGIT, B7-H3, and B7-H4—have emerged as promising candidates and are currently under active investigation in TNBC. In this section, we focus on the immunological functions of these alternative immune checkpoints and systematically summarize the preclinical evidence and clinical progress of their corresponding inhibitory strategies [20,87].

### 3.1. CTLA-4

Cytotoxic T-lymphocyte antigen 4 (CTLA-4), also known as CD152, is a key immune checkpoint that is predominantly localized within intracellular vesicles of FoxP3^+^ (Forkhead Box P3) Tregs and activated T cells, where it functions to inhibit T cell activation [88,89]. In the early stages of T cell activation, CD28 on the surface of T cells binds to two B7 family ligands, CD80 (B7-1) and CD86 (B7-2), expressed on APCs. This interaction provides a co-stimulatory signal that activates the TCR signaling pathway and promotes T cell activation. As T cells become activated, CTLA-4 expression is upregulated and competes with CD28 for binding to CD80 and CD86. Notably, CTLA-4 exhibits a significantly higher binding affinity for B7 ligands than CD28, thereby effectively attenuating CD28 mediated costimulatory signaling and limiting T cell activation and proliferation, thereby exerting a negative regulatory effect on immune responses [90,91].

In TNBC, tumor cells may exploit this mechanism to enhance CTLA-4 mediated immunosuppression, thereby suppressing antitumor T cell activity and promoting immune escape. Moreover, CTLA-4 plays a central role in the suppressive function of intratumoral FoxP3^+^ Tregs, which are frequently enriched within the TME. These infiltrating Tregs mediate immune suppression through multiple mechanisms, including the activation of immunosuppressive and tumor-promoting signaling pathways, ultimately inhibiting effective antitumor immune responses [80,92,93].

CTLA-4 blockade primarily reduces CTLA-4 mediated transcytosis of CD80/CD86, enhances CD28 mediated co-stimulatory signaling, restores T cells activation while suppressing Treg function, and ultimately promotes the immune-mediated killing of tumor cells [94,95]. In addition, anti-CTLA-4 antibodies can induce antibody-dependent cellular cytotoxicity (ADCC) mediated by tumor-associated macrophages. While PD-1/PD-L1 inhibitors mainly regulate CD8^+^ T cell effector function, CTLA-4 blockade modulates clonal expansion of CD4^+^ T cell. Consequently, their combination provides complementary immune activation and may result in enhanced antitumor efficacy [96,97].

### 3.2. LAG-3

Lymphocyte activation gene 3 (LAG-3), also known as CD223, is a structural homolog of CD4 and functions as an inhibitory immune checkpoint receptor. It is primarily expressed on activated CD4^+^ and CD8^+^ T cells, B cells, NK cells, and DCs, and is highly upregulated on exhausted T cells. LAG-3 negatively regulates T cell function within the TME, thereby facilitating tumor immune escape [98,99,100]. LAG-3 can interact with multiple ligands, the most well-characterized of which is the MHC-II molecule. Owing to its high structural homology with CD4, LAG-3 competes with CD4 for binding to MHC-II; however, LAG-3 exhibits a higher binding affinity for MHC-II than CD4. This interaction negatively regulates CD4^+^ T cell activation, thereby contributing to immunosuppression within the TME [101].

In addition to regulating CD4^+^ T cells, LAG-3 can also limit the activation of CD8^+^ T cells. Upon TCR stimulation, LAG-3 can associate with the TCR–CD3 complex in cis and localize to the immunological synapse. Within the synapse, Lck is phosphorylated and dissociates from the CD4/CD8 co-receptor, which restricts ZAP70 phosphorylation, thereby inhibiting TCR signaling and suppressing the activation of both CD4^+^ and CD8^+^ T cells. In addition, the cytoplasmic domain of LAG-3 contains a conserved KIEELE motif, which has been reported to inhibit calcium channel activity, leading to reduced intracellular Ca^2+^ levels and subsequent suppression of T cell activation and effector function [102,103]. Given its role in restraining T cell activation and promoting immune evasion, LAG-3 represents a promising target for tumor immunotherapy.

Anti-LAG-3 ICIs can block the LAG-3/MHC-II interaction, thereby restoring T cell and overall immune cell function. In TNBC patients, elevated expression levels of LAG-3 and PD-L1 have been frequently observed, suggesting the presence of overlapping yet non-redundant inhibitory pathways within the tumor microenvironment. Accordingly, simultaneous blockade of the PD-1/PD-L1 and LAG-3 pathways has demonstrated enhanced antitumor activity in preclinical models and early clinical studies [104,105]. Currently, multiple anti-LAG-3 ICIs, as well as combination therapies targeting both LAG-3 and the PD-1/PD-L1 axis, are being evaluated in clinical trials for cancer treatment, highlighting LAG-3 as a promising next-generation immune checkpoint target for overcoming resistance to PD-1/PD-L1–based immunotherapy [106].

### 3.3. TIM-3

T cell immunoglobulin and mucin-domain containing protein 3 (TIM-3), also known as CD366 or HAVCR2, is a member of the *TIM* gene family and is expressed on multiple immune cell subsets, including CD4^+^ T helper 1 (Th1), NK cells, macrophages, and DCs, as well as on certain tumor cells that secrete IFN-γ. TIM-3 has been identified as a key regulator of CD8^+^ T cell exhaustion in cancer. TIM-3 interacts with multiple ligands, including galectin-9 (Gal-9), phosphatidylserine (PtdSer), high-mobility group protein B1 (HMGB1), and carcinoembryonic antigen-related cell adhesion molecule 1 (CEACAM-1). Structural and biochemical studies have demonstrated that the immunoglobulin variable (IgV) domain of TIM-3 serves as the principal ligand-binding region. Notably, multiple ligands, including CEACAM-1, HMGB1, and PtdSer, engage the FG–CC′ loop within the IgV domain of TIM-3 [102,107].

Among these, Gal-9 was the first identified ligand for TIM-3, and Gal-9/TIM-3 interaction induces intracellular calcium influx in Th1 cells, leading to Th1 apoptosis and contributing to immune tolerance. In addition, TIM-3 can form heterodimers with CEACAM-1, further inhibiting T cells activation and promoting immune tolerance [108,109]. PtdSer, which is exposed on the surface of apoptotic cells, can bind to TIM-3 and trigger phagocytic signaling in APCs. This interaction modulates TCR activation and IL-2 production, while suppressing the NF-κB and PI3K-AKT-mTOR signaling pathways, ultimately leading to reduced cytotoxic activity of NK cells and contributing to immune tolerance [110,111]. Additionally, TIM-3 is broadly expressed on DCs and can bind to HMGB1. This interaction interferes with the delivery of nucleic acids into DC endosomal vesicles, suppressing nucleic acid mediated innate immune activation and impairing innate immune cell function [112,113]. Both CEACAM-1 and HMGB-1 mediated TIM-3 signaling converge on the inhibition of NF-κB activation, and further reinforcing an immunosuppressive tumor microenvironment [114].

The FG–CC′ loop has emerged as a critical structural epitope and a promising therapeutic target for immune checkpoint blockade strategies directed against TIM-3. TIM-3 blockade primarily exerts their antitumor effects by blocking the interaction between TIM-3 and its cognate ligands, thereby relieving TIM-3–mediated inhibitory signaling and facilitating the restoration of immune cell function. This blockade has been associated with reactivation of key downstream pathways, including NF-κB and PI3K–AKT–mTOR signaling. Given its role in T cell exhaustion, TIM-3 represents a key complementary target to PD-1, and combined inhibition of TIM-3 and PD-1 provides a rational strategy to enhance CD8^+^ T cell–mediated antitumor immunity [99,114,115].

### 3.4. TIGIT

T cell immunoreceptor with immunoglobulin and ITIM domain (TIGIT), also known as Vsig9, Vstm3, or WUCAM, is an inhibitory receptor that can be transiently upregulated on T cells following TCR stimulation. TIGIT is expressed on various immune cells, including CD8^+^ and CD4^+^ T cells; NK cells; Tregs; and follicular helper T cells. TIGIT binds to multiple ligands expressed on APCs, such as CD155 (PVR), CD112 (PVRL2), and CD113 (NECTIN-3). Notably, CD155 and CD112 also serve as ligands for the co-stimulatory receptor CD226 (DNAM-1), placing TIGIT within a complex co-stimulatory and co-inhibitory signaling network analogous to the CD28/CTLA-4/CD80/CD86 axis [99,102,116].

Among these interactions, CD155 exhibits the highest affinity for TIGIT compared with CD226 and CD96. Upon binding to its ligand CD155, TIGIT undergoes phosphorylation of its ITIM like motif, which enables the recruitment of the adaptor protein Grb2. This interaction subsequently recruits the SH2 domain–containing inositol-5-phosphatase 1 (SHIP1), leading to the inhibition of multiple downstream signaling pathways, including the PI3K, MAPK, and NF-κB pathways [117]. In addition, TIGIT has been reported to suppress NK cell function by inhibiting CD155-induced phosphorylation of ERK1/2 as well as ZAP70/Syk, thereby attenuating activating signaling cascades in NK cells [118]. The engagement of TIGIT with CD155 on DCs promotes the development of tolerogenic DCs, reduces IL-12 production, and increases IL-10 secretion, thereby impairing T cell proliferation and suppressing IFN-γ production by effector T cells [119,120].

In addition, TIGIT binds to CD155 with higher affinity than CD226, thereby limiting CD226-mediated co-stimulatory signaling. Investigations have demonstrated that CD226 plays a critical role in regulating TCR-mediated T cell activation and function. Consequently, the preferential binding of TIGIT to CD155 inhibits CD226-dependent co-stimulation, resulting in indirect suppression of T cell activity and impairment of antitumor immune responses. Beyond ligand competition, TIGIT can directly interact with CD226 in cis, disrupting CD226 homodimerization and further limiting its ability to engage CD155 [102,117].

TIGIT is widely recognized as a marker of exhausted T cells within the TME. Therapeutic blockade of TIGIT primarily functions by disrupting the inhibitory interaction between TIGIT and its ligand CD155, thereby reshaping cytokine secretion profiles, reversing T- and NK-cell exhaustion, and restoring effective antitumor immune responses [121]. Similarly to LAG-3, therapeutic strategies targeting TIGIT-including monoclonal antibodies and PD-1/TIGIT bispecific immune checkpoint inhibitors—have demonstrated the potential to enhance CD8^+^ T cell activity. These approaches are currently under clinical evaluation, particularly in tumors that exhibit resistance to PD-1-based immunotherapies [102,122,123].

### 3.5. Other Immune Checkpoints in the B7 Family

The B7 family, as key co-stimulatory and co-inhibitory molecules in the immune system, provides both positive signals to activate T cell function and negative signals to suppress it. Currently, the B7 family comprises several members, including B7-1, B7-2, B7-DC, B7-H1, B7-H2, B7-H3, B7-H4, B7-H5, B7-H6, and B7-H7 [124,125]. The preceding sections have introduced B7-1 (CD80) and B7-2 (CD86), which bind to CTLA-4, and B7-H1(PD-L1), which interacts with PD-1. Studies have shown that among other B7 family immune checkpoints, B7-H3 and B7-H4 are highly expressed in TNBC and represent promising targets for immunotherapy. Therefore, this section focuses on the roles and mechanisms of B7-H3 and B7-H4 within the B7 family [126].

#### 3.5.1. B7-H3

B7-H3, also known as CD276, is a type I transmembrane protein expressed on immune cells, containing APCs, NK cells, B cells, and T cells. In normal tissues, B7-H3 expression is low; however, it is highly expressed in a variety of cancers, such as breast cancer, gastric cancer, colorectal cancer, and lung cancer, making it a promising target for tumor immunotherapy. Functioning as a co-stimulatory molecule, B7-H3 can promote the proliferation of CD4^+^ and CD8^+^ T cells, enhance cytotoxic T cell activity, and stimulate IFN-γ production via TCR signaling, thereby exerting immune-stimulatory effects [127,128]. However, B7-H3 also exhibits immunosuppressive and tumor-promoting roles in the TME, limiting T cell infiltration and promoting CD8^+^ T cell exhaustion, thereby suppressing anti-tumor immunity and facilitating immune evasion. In addition, B7-H3 can promote the polarization of macrophages toward the M2 phenotype and impair DCs function, further contributing to an immunosuppressive microenvironment [129,130]. Notably, in TNBC, high B7-H3 expression on TAMs has been reported to hinder CD8^+^ T cell infiltration by remodeling the extracellular matrix and inducing aberrant angiogenesis, thereby reinforcing immune suppression [131]. whereas blockade of B7-H3 enhances intratumoral infiltration of CD8^+^ T cells and NK cells, while simultaneously suppressing tumor cell proliferation.

Consistent with its immunosuppressive functions, B7-H3 has been shown to inhibit T cell–mediated antitumor immunity, whereas blockade of B7-H3 enhance intratumoral infiltration of CD8^+^ T cells and NK cells, while simultaneously suppressing tumor cell proliferation. Currently, numerous anti-B7-H3 immunotherapeutic strategies are under development, including monoclonal antibodies, bispecific antibodies, ADCs, and CAR-T cell therapies, many of which have entered clinical trials for cancer treatment [132,133]. Importantly, combination therapeutic strategies incorporating B7-H3 blockade may represent a promising direction for future research. Preclinical studies have demonstrated that B7-H3 inhibition in combination with other immune checkpoint inhibitors, including PD-1 and CTLA-4 blockade, can elicit enhanced antitumor immune responses, highlighting the potential clinical value of such combinatorial approaches [134].

#### 3.5.2. B7-H4

B7-H4, also known as VTCN1, is a type I transmembrane protein belonging to the B7 family. B7-H4 mRNA is widely expressed across various tissues, whereas its protein expression is not expressed or relatively low in normal healthy tissues. In contrast, B7-H4 is highly expressed in numerous solid tumors, containing breast cancer, ovarian cancer, and endometrial cancer. Similarly to B7-H3, B7-H4 primarily functions as an immunosuppressive immune checkpoint molecule, although a specific receptor for B7-H4 has yet to be identified [135,136]. B7-H4 mediates immunosuppression by inhibiting T cell function. It suppresses both CD4^+^ and CD8^+^ T cell responses, inhibits T cell proliferation, as well reduces the production of key cytokines, including IFN-γ and IL-2 [137]. Simultaneously, B7-H4 promotes an immunosuppressive TME driving T cell exhaustion, increasing Treg infiltration, and recruiting TAMs, thereby facilitating tumor progression [138]. Consistent with these immunosuppressive effects, B7-H4 overexpression in tumors enhances proliferation, migration, and invasion, and is associated with clinical parameters such as tumor size, lymph node metastasis, and overall patient survival [139,140].

Blockade of B7-H4 alleviates B7-H4–mediated immunosuppression of T cells, thereby restoring antitumor immune responses [141]. Current cancer therapies targeting B7-H4 include monoclonal antibodies, bispecific antibodies, and ADCs, which have demonstrated efficacy in preclinical models and are now undergoing clinical evaluation. Studies have shown that B7-H4 expression is inversely correlated with PD-L1 expression, suggesting that anti-B7-H4 therapies may benefit patients who are PD-L1 negative or nonresponsive to PD-L1 based treatments [140,142].

Taken together, these distinct features position B7-H3 and B7-H4 as attractive next-generation immune checkpoint targets, particularly for combination strategies aimed at overcoming resistance to PD-1/PD-L1–based immunotherapy in TNBC.

**Figure 2:**
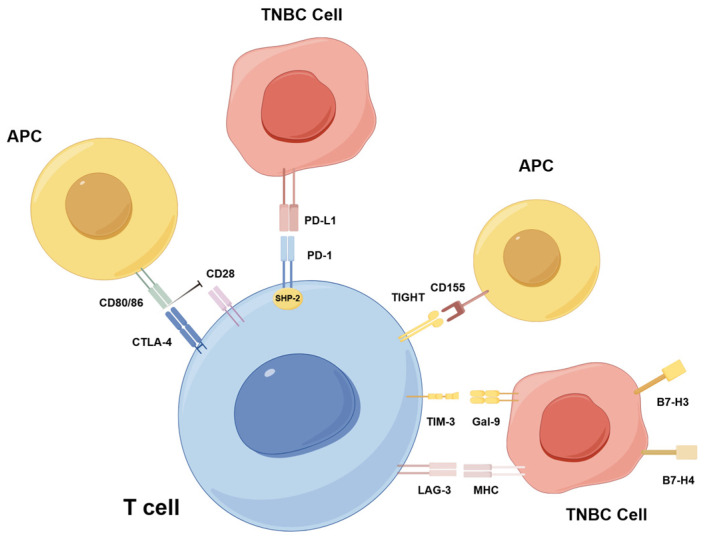
Immune checkpoint pathways regulating T cell activity in the TNBC TME. Numerous immune checkpoints and their corresponding ligands, including classical immune checkpoints PD-1/PD-L1, CTLA-4/B7 (CD80/CD86) and emerging exhaustion-associated receptors LAG-3/MHC-II, TIM-3/Gal-9, and TIGIT/CD155 interactions regulate T cell activity. B7-H3 and B7-H4 are also highly expressed in TNBC. The figure was drawn by Figdraw. TNBC—Triple-Negative Breast Cancer; APC—Antigen-Presenting Cell; MHC—Major Histocompatibility Complex.

The immune checkpoint pathways that regulate T cell activity in the TNBC TME are illustrated in Figure 2.

### 3.6. Clinical Applications of Immune Checkpoint Inhibitors Targeting Alternative Immune Checkpoints

Recently, an increasing number of potential immune targets in TNBC have been explored. In addition to PD-1/PD-L1, CTLA-4 is one of the most widely studied targets, and CTLA-4 inhibitors are currently being evaluated in clinical trials for TNBC. Ipilimumab, the first CTLA-4 inhibitor approved for clinical use, has demonstrated efficacy in melanoma, colorectal cancer, and hepatocellular carcinoma. However, the therapeutic benefit of ipilimumab monotherapy is limited, and combination strategies have been shown to enhance its antitumor activity. At present, multiple phase II clinical trials are investigating ipilimumab in combination with radiotherapy, chemotherapy, or PD-1/PD-L1 inhibitors for the treatment of TNBC [143,144]. In addition, another CTLA-4 inhibitor, tremelimumab, has also entered clinical trials for TNBC. A recent study evaluating the combination of durvalumab and tremelimumab, anti-CTLA-4 combined with anti-PD-L1, with chemotherapy demonstrated only limited anti-tumor activity in patients with pretreated advanced breast cancer. Therefore, novel combination strategies are still needed to improve therapeutic efficacy [145,146]. However, anti-CTLA-4 therapy has not yet been approved for any subtype of breast cancer, and further investigation exploring its combination with other therapeutic modalities is warranted.

Compared with PD-1/PD-L1 and CTLA-4 inhibitors that have already been approved for clinical application in cancer treatment, most monoclonal antibodies targeting alternative immune checkpoints remain under clinical investigation. Emerging immune checkpoints, including LAG-3, TIM-3, and TIGIT—key mediators of T-cell exhaustion—as well as B7 family members such as B7-H3 and B7-H4, which contribute to tumor immune evasion, represent promising therapeutic targets in TNBC and may help overcome resistance to PD-1/PD-L1 blockade. However, most corresponding therapeutic antibodies remain in early-phase clinical development and have not yet received regulatory approval for TNBC treatment. In 2022, the anti-LAG-3 antibody relatlimab (BMS-986016), in combination with nivolumab, was approved by the FDA for the treatment of advanced melanoma, marking the first LAG-3 immune checkpoint inhibitor to receive regulatory approval [147]. In addition, ieramilimab (LAG525), a monoclonal antibody targeting LAG-3, inhibits the interaction between LAG-3 and MHC-II molecules. In TNBC, a phase II clinical trial is currently evaluating the safety and efficacy of combining LAG525 with the anti-PD-1 antibody spartalizumab and platinum-based chemotherapy to achieve dual blockade of LAG-3 and PD-1. The ORR was 14.3% in patients who had not received prior anti-PD-1/PD-L1 therapy and were treated every three weeks, and 4.8% in those treated every four weeks. In contrast, no clinical responses were observed in TNBC patients who had received prior anti-PD-1/PD-L1 treatment [106,148,149].

INCAGN02390 is a novel, fully human, Fc-engineered, Aglycosylated (N297A) anti-TIM-3 IgG1κ monoclonal antibody that is currently being evaluated in a phase I clinical trial for advanced solid tumors, including TNBC [150]. Tiragolumab is an anti-TIGIT antibody that enhances T cell-mediated anti-tumor immunity by blocking TIGIT. When combined with anti-PD-1/PD-L1 therapies, TIGIT blocking alleviates CD155 mediated suppression, thereby amplifying PD-1/PD-L1 mediated T cell activation [151]. Clinical trials have demonstrated that the combination of tiragolumab, the anti-PD-1 antibody atezolizumab, and chemotherapy has advanced to phase II for the treatment of TNBC. However, TIM-3 and TIGIT targeted therapies in TNBC remain in the early clinical stages and warrant further investigation, particularly in combination with other therapeutic approaches [152].

For B7 family immune checkpoints, although there are currently no approved ICIs targeting B7-H3 and B7-H4 for the treatment of TNBC, and limited clinical trials have been conducted, their high expression and immunosuppressive properties in TNBC position them as promising therapeutic targets [140,153].

Clinical trials involving monoclonal antibodies targeting alternative immune checkpoints in TNBC are summarized in Table 4. In addition to the clinically established PD-1/PD-L1 and CTLA-4 pathways, emerging inhibitory immune checkpoints associated with T cell exhaustion, including LAG-3, TIM-3, and TIGIT, have attracted increasing attention as potential therapeutic targets in TNBC. Although currently available clinical evidence remains limited and most studies are still in early-phase, these alternative ICIs are being actively investigated either as monotherapy or in combination with anti-PD-1/PD-L1 antibodies. Such combination strategies are based on the rationale that compensatory upregulation of alternative immune checkpoints may contribute to adaptive resistance following PD-1/PD-L1 blockade.

### 3.7. Bispecific Antibody

Complex diseases such as malignant cancer, which involve multiple molecules and intricate regulatory mechanisms, monoclonal antibodies targeting individual immune checkpoints often exhibit limited efficacy and are associated with numerous adverse reactions. In recent decades, significant progress has been made in the development of bispecific antibodies (BsAbs). BsAbs are engineered recombinant proteins designed to simultaneously bind two distinct antigens or antigenic epitopes, offering the potential for enhanced therapeutic outcomes [39]. By simultaneously targeting two distinct signal transduction pathways, BsAbs offer enhanced specificity for binding to tumor cells and reduced toxicity. Compared to conventional combination therapies involving monoclonal antibodies, BsAbs also present the advantage of potentially lower treatment costs.

Currently, BsAbs targeting immune checkpoints are under development for the treatment of TNBC and have entered clinical trials. Multiple preclinical and clinical studies have explored the use of bispecific antibodies targeting PD-1/PD-L1 for immunotherapy in TNBC. KN046 is a PD-L1/CTLA-4 bispecific single-domain antibody Fc fusion protein. Dual blockade of CTLA-4 and PD-L1 enhances CD28-dependent co-stimulatory signaling and restores T cell function, while promoting the depletion of intratumoral Treg cells, collectively contributing to improved antitumor immune responses. Preclinical studies have demonstrated that KN046 elicits superior T cell activation compared to PD-1/PD-L1 monotherapy or combination therapy involving PD-1/PD-L1 and CTLA-4. In a clinical trial evaluating KN046 in combination with albumin-bound paclitaxel in patients with locally advanced unresectable or metastatic TNBC, an ORR of 44.0% was observed in 25 evaluable patients, with a mDoR that remains immature. The mPFS was 7.33 months, and the mOS was 30.92 months. Notably, PD-L1-positive patients exhibited a PFS of 8.61 months and a 2-year OS rate of 62.5%, whereas PD-L1-negative patients had a PFS of 4.73 months and a 2-year OS rate of 57.1%. These results suggest that TNBC patients tolerate the combination therapy well, demonstrating the promising anti-tumor efficacy of KN046 [154]. MGD013 is a novel molecule engineered to synergistically block PD-1 and LAG-3, demonstrating antitumor activity and an acceptable safety profile in TNBC patients [155]. BNT327 (Pumitamig), which targets both PD-L1 and VEGF-A, inhibits tumor immune escape and angiogenesis, demonstrating significant efficacy in small cell carcinoma patients. A recent phase III clinical trial in TNBC patients is investigating PFS and OS.

In addition, several innovative BsAbs targeting PD-1/PD-L1 have completed preclinical evaluations and are expected to further assess their safety and efficacy in upcoming clinical studies. For instance, BiTP, a TGF-β/PD-L1 BsAb, was found to effectively inhibit TGF-β-Smad and PD-L1-NFAT signaling in in vitro and in vivo experiments on TNBC, thus exhibiting antitumor activity [156]; ATG-101 (Zr-Df-ATG-101) is a BsAb targeting PD-L1 and 4-1BB. In vivo studies have demonstrated its antitumor activity in MDA-MB-231 xenograft mice expressing PD-L1 [157]; in addition, researchers developed a BsAb by coupling a human-derived IgG1 monoclonal antibody targeting PD-L1 with a humanized camelid VHH nanobody against B7-H3. This B7-H3 × PD-L1 BsAb (IgG1-VHH) targets both PD-1 and B7-H3. The results demonstrated that, compared to monotherapy with individual monoclonal antibodies, the BsAb enhanced specificity against double-positive B7-H3 and PD-L1 tumors, synergistically boosting the anti-tumor efficacy in TNBC [158].

Additionally, numerous BsAbs targeting other immune checkpoints are under clinical trials [159]. Such as JK08, which targets both CTLA-4 and IL-15, and XmAb22841, which targets both CTLA-4 and LAG-3, have both demonstrated safety and tolerability as monotherapies or in combination therapies in patients with solid tumors, including TNBC. Meanwhile, for B7 family immune checkpoints, preclinical studies have shown that the B7-H3-CD3 bispecific antibody TAK-280 can reprogram the TME, block immune checkpoint signaling pathways, and significantly enhance the efficacy of immunotherapy in TNBC. In addition, B7-H4/CD3 BsAbs constructed based on Fab and scFv structures have been shown to exhibit potent cytotoxic activity against B7-H4^+^ TNBC cell lines and also possess in vivo antitumor activity [160].

Clinical trials involving BsAbs targeting immune checkpoints in TNBC are summarized in Table 5. Among the currently investigated BsAbs, some simultaneously target two immune checkpoint molecules, whereas others combine immune checkpoint blockade with tumor-associated or immunomodulatory targets. Compared with conventional monoclonal antibodies, BsAbs may provide enhanced antitumor activity through dual-target immune modulation and synergistic activation of antitumor immune responses. In addition, compared with combination regimens involving multiple monoclonal antibodies, BsAbs may offer potential advantages in treatment convenience and toxicity management. However, most currently available evidence remains preliminary, and further clinical validation is required to determine their long-term efficacy and safety in TNBC treatment.

## 4. Emerging Immune Checkpoint-Based Immunotherapy in TNBC

ICB therapy, represented by PD-1/PD-L1 inhibitors, has demonstrated significant clinical value in the treatment of TNBC and has become a key component of TNBC immunotherapy. Over the past few years, various emerging immunotherapeutic strategies have been developed. This section primarily summarizes the research progress and applications of other immune checkpoint targeted immunotherapies in TNBC, including ADCs, CAR-T therapy, tumor vaccines, and OVs [62].

### 4.1. Overview of ADC

ADCs are a novel class of targeted therapeutics, consisting of three key components: a tumor-targeting monoclonal antibody, a cytotoxic payload, and a linker that conjugate the two components. By utilizing recombinant monoclonal antibodies that target tumor cell surface antigens, ADCs can effectively deliver cytotoxic agents directly to the TME, thereby minimizing off-target systemic toxicity [161]. For TNBC, Sacituzumab govitecan (SG) is a first-in-class Trop-2 ADC, formed by conjugating a Trop-2 monoclonal antibody with a cleavable SN-38 linker. In 2021, the FDA approved SG for patients with unresectable locally advanced or metastatic TNBC who have previously received two or more systemic therapies, including at least one for metastatic disease. SG is the first ADC to significantly improve OS in metastatic TNBC patients [7,162]. Currently, there are reports of using immune checkpoints as targets for ADCs in the immunotherapy of TNBC.

Although ADCs primarily function through targeted cytotoxic mechanisms rather than direct immune activation, ADCs targeting immune checkpoint molecules such as B7-H3, B7-H4, and PD-L1, which are highly expressed in tumors, possess both cytotoxic and immunomodulatory activities. Therefore, these agents are often considered immune checkpoint-related therapeutic tools. This dual approach aims to enhance tumor-specific immune activation while delivering targeted therapy directly to the TME, thereby minimizing off-target toxicity. Currently, preclinical studies have demonstrated their potential to overcome tumor heterogeneity and treatment resistance, highlighting their promising therapeutic efficacy in TNBC by addressing resistance mechanisms that limit the effectiveness of traditional therapies [163].

### 4.2. Research and Application of Immune Checkpoint Targeted ADCs in TNBC

Several immune checkpoint-related ADCs are currently being evaluated in preclinical and early-phase clinical studies for TNBC; however, none have yet received clinical approval for TNBC therapy. In particular, B7-H3 and B7-H4 are highly expressed in TNBC cells but show limited expression in normal tissues, making them promising and selective targets for ADCs. For instance, m276-SL-PBD, a modified talirine PBD-based fully human B7-H3 ADC, has demonstrated in preclinical studies the ability to eradicate 500–1000 mm^3^ TNBC xenografts at doses 10 to 40 times lower than the maximum tolerated dose; MGC018, a B7-H3-targeting ADC, utilizes a cleavable linker-duocarmycin payload, valine-citrulline-seco duocarmycin hydroxybenzamide azaindole (vc-seco-DUBA), conjugated to a humanized anti-B7-H3 IgG1/kappa monoclonal antibody via reduced interchain disulfides, with an average DAR of approximately 2.7. While MGC018 showed promising anti-tumor activity in preclinical studies, recent clinical trials were halted due to patient fatalities [164,165].

Besides B7-H3, B7-H4, another member of the B7 family, has emerged as a prominent target for ADCs in recent years. Several ADCs targeting B7-H4 have entered clinical trials for the treatment of TNBC. AZD8205 and BG-C9074 are B7-H4-targeted ADCs with a topoisomerase I inhibitor payload. XMT-1660 is a novel site-specific DAR6 ADC targeting B7-H4. LNCB74 is a B7-H4 antibody conjugated to the microtubule-damaging payload monomethylostatin E, with a DAR of 4. All of these ADCs have demonstrated preliminary clinical potential in TNBC patients [136,166,167,168].

However, ADCs also have limitations. Increased drug resistance and cumulative toxicity can impact their clinical efficacy. TNBC, characterized by high relapse rates, frequent metastasis, and a strong propensity for developing drug resistance, poses a particular challenge [169]. Most TNBC patients may not benefit from ADCs that carry a single cytotoxic payload. Recent studies have highlighted the ongoing investigation of several innovative ADCs. Preclinical studies over the past few years have demonstrated the potential of dual-load ADCs, such as those combining toxic payloads and immunomodulatory Toll-like receptor 7/8 (TLR7/8) agonists link with B7-H3 monoclonal antibodies, for the treatment of TNBC [170]. Emerging ADCs, such as dual-targeting bispecific ADCs, are also being explored. One such example is DB-1419, a bispecific ADC that conjugates B7-H3 × PD-L1 with a novel topoisomerase I inhibitor, P1003, achieving a DAR of 8. This conjugate has demonstrated effective antitumor activity, particularly in PD-L1-resistant tumors, where it shows significant tumor growth inhibition [163].

### 4.3. Overview of CAR-T Cell Therapy

CAR-T therapy is a targeted immunotherapy based on live cells. The process begins with the collection of peripheral blood mononuclear cells (PBMCs) from the patient (autologous CAR-T) or a healthy donor (allogeneic CAR-T). T lymphocytes are isolated from the PBMCs and genetically engineered to express chimeric antigen receptors (CARs) on their surface. These modified cells are then infused back into the patient, where they specifically recognize target antigens, rapidly proliferate in vivo, and eliminate tumor cells [171,172]. CAR is the core component of CAR-T therapy, enabling T cells to recognize tumor antigens in an HLA-independent manner, which allows CAR-T cells to target a broader range of antigens compared to natural TCRs, thereby enhancing their capacity to specifically identify and eliminate tumor cells [173].

CAR-T therapy offers advantages such as robust in vivo expansion and a relatively rapid preparation process. It has already achieved significant success in treating relapsed B-cell hematologic malignancies. However, the development of CAR-T therapy for solid tumors faces significant challenges, including TME immunosuppression, limited antigen-specific recognition, tumor heterogeneity, and CAR-T cells depletion [40,174,175]. Beyond CAR-T therapy, NK cells, macrophages, and mesenchymal stem cells (MSCs) can also be engineered for CAR-based immunotherapy in treatment of malignant tumor [176].

### 4.4. Research and Application of Immune Checkpoint Targeted CAR-T Cell Therapy in TNBC

CAR-T therapy has shown promising clinical potential in TNBC, although current evidence is primarily derived from preclinical studies and early-phase clinical trials. In addition, the identification of appropriate target antigens remains critical for improving its therapeutic efficacy and safety. PD-1/PD-L1, the most prominent immune checkpoint, has emerged as a key target for CAR-T cell therapy. Preclinical studies suggest PD-L1-targeted CAR-T cell therapy is believed to block the PD-1/PD-L1 axis, inhibiting TNBC growth in vivo in mouse models [177].

Furthermore, B7 family B7-H3 is highly expressed in TNBC, and in recent years, several novel CAR-T therapies targeting B7-H3 have entered preclinical studies. Results suggest that constructing a CAR-T cell line via lentiviral transduction, which targets B7-H3 and incorporates 4-1BB co-stimulatory molecules along with STAT3 and STAT5 related activation motifs, provides an effective strategy for treating TNBC [178]. Additionally, studies have designed optimized bispecific B7-H3 and CSPG4 CAR-T cells, demonstrating their ability to eradicate tumors with mixed antigen expression in a TNBC patient-derived xenograft (PDX) model [179]; ICOS-enhanced B7-H3-CAR-T cells (ICOS-B7-H3-CAR), characterized by amplified ICOS expression and targeting of the B7-H3 antigen, have demonstrated antitumor activity against TNBC cells both in vitro and in vivo [180]. CAR-T cells targeting B7-H3 have entered clinical trials. The iC9-CAR, a T lymphocyte chimeric antigen receptor targeting the B7-H3 antigen, is currently being investigated in a dose-escalation study for safety in patients with relapsed/refractory TNBC.

### 4.5. Overview of Tumor Vaccine

Cancer vaccine therapy is an emerging immunotherapeutic strategy that introduces tumor antigens into the patient to activate or enhance the immune system’s ability to recognize and eliminate tumor cells, with the potential to convert immunologically “cold” tumors into “hot” tumors by promoting antigen presentation and T cell infiltration. Mechanistically, APCs, such as DCs and macrophages, capture and process these antigens, presenting them via MHC-I and MHC-II molecules. This presentation activates CD8^+^ cytotoxic T lymphocytes and CD4^+^ helper T cells, thereby inducing robust antitumor immune responses [181,182,183]. There are numerous types of tumor vaccines, including cell-based vaccines (such as whole tumor cell and DC vaccines), protein-based vaccines (such as full-length proteins and peptide vaccines), nucleic acid-based vaccines (including DNA and mRNA vaccines), and vector-based vaccines (such as bacterial and viral vector vaccines) [184].

Tumor antigens used in cancer vaccines can be broadly categorized into two types: TAAs and tumor-specific antigens (TSAs). TAAs are antigens that are expressed at low levels in normal tissues but are overexpressed in tumor cells. Representative examples include HER2, p53, and MUC1. Whereas TSAs are neoantigens that arise from tumor-specific mutations and are expressed exclusively in tumor tissues, but not in normal cells [185,186,187]. Designing neoantigen-based tumor vaccines using TSAs has emerged as one of the most promising strategies in cancer immunotherapy. Since tumor-specific neoantigens are not expressed in normal tissues, they can effectively evade central immune tolerance. Consequently, vaccines targeting neoantigens can elicit more robust and specific immune responses than those targeting TAAs, leading to the activation of neoantigen-specific T lymphocytes and enabling the development of personalized cancer vaccines [188].

### 4.6. Research and Application of Immune Checkpoint Targeted Tumor Vaccine in TNBC

Regarding tumor vaccine research targeting immune checkpoints in TNBC, studies are still in the preclinical stage. Given that PD-1/PD-L1 is expressed at very low levels in normal tissues but highly expressed in tumor tissues, it has been identified as an ideal target for tumor vaccine development [189]. IMU-201 is a PD-1 B cell epitope peptide vaccine that has demonstrated anti-tumor efficacy comparable to that of ICIs, significantly suppressing tumor growth in a BALB/c mouse 4T1 tumor-bearing mice model [190]. Currently, clinical trials of IMU-201 in patients with non-small cell lung cancer have demonstrated a favorable safety profile. Its potential application in TNBC may therefore hold promising translational value for future clinical development.

Currently, research on cancer therapeutic vaccines remains limited and is still in the early stages of development. In 2010, the FDA approved sipuleucel-T for the treatment of hormone-refractory advanced prostate cancer, marking the first—and, to date, the only—cell-based immunotherapy approved for solid tumors. Over the past few years, the rapid advancement of therapeutic vaccines for melanoma has further laid the groundwork for the future of cancer vaccines targeting other malignant tumors [191]. In TNBC, DC–based vaccines and neoantigen tumor vaccines in combination with ICIs have entered clinical trials. Although tumor vaccines have not yet been widely tested in clinical trials for TNBC, current preclinical studies and progress in other tumor types indicate that tumor vaccines hold promising potential in TNBC. Further in-depth research is warranted to fully explore their therapeutic efficacy in this setting.

### 4.7. Overview of Oncolytic Virus

OVs represent a novel form of cancer immunotherapy. Their mechanism of action involves the selective replication of viruses within tumor cells, leading to tumor cell lysis and the release of TAAs, thereby inducing antitumor immune responses in the host. OVs are distinguished by their potent replicative capacity and relatively low toxicity toward normal cells [192]. There are two main types of OVs, including naturally occurring viruses and genetically engineered viruses. Because tumor cells often exhibit impaired antiviral defenses resulting from abnormal cytokine expression, they are particularly susceptible to OVs infection and subsequent oncolysis [193]. Talimogene laherparepvec (T-VEC) is a genetically modified type I herpes simplex virus engineered to express granulocyte-macrophage colony stimulating factor (GM-CSF), which enhances systemic antitumor immune responses. It has been approved by the FDA as the first OV therapy for the treatment of patients with metastatic melanoma [194,195]. In TNBC, clinical trials have shown that T-VEC combined with neoadjuvant chemotherapy can reduce the residual cancer burden (RCB) index in patients, thereby enhancing therapeutic efficacy and advancing the clinical development of OV based therapies in TNBC [196].

### 4.8. Research and Application of Immune Checkpoint Targeted Overview of Oncolytic in TNBC

At present, the evaluation of OV targeting immune checkpoints in TNBC remains largely confined to the preclinical stage. For instance, combination therapy involving five OVs engineered to express IL-12, IL-15, GM-CSF, PD-1v, and IL-7 × CCL19 demonstrated potent antitumor effects in the 4T1 TNBC tumor-bearing mice model [197]. Furthermore, VG161, an OV engineered to express IL-12, IL-15 along with its receptor α subunit (IL-15RA), and a PD-L1-blocking fusion protein (PD-L1B), has been shown to inhibit tumor growth and elicit a robust antitumor immune response in a TNBC tumor-bearing mice model. Moreover, when combined with paclitaxel, VG161 further enhances antitumor immunity and significantly reduces lung metastases in TNBC [198]. In addition, CF33-hNIS-anti-PD-L1 is an oncolytic chimeric vaccinia virus engineered to express two transgenes: the human sodium iodide symporter (hNIS) and a single-chain variable fragment (scFv) targeting PD-L1. In vivo studies have demonstrated its potent tumor growth inhibition in TNBC bearing mice, providing a strong rationale for future first-in-human clinical trials in TNBC patients [199].

Currently, the clinical application of OVs remains limited; however, multiple trials evaluating novel OVs in combination with chemotherapy, radiotherapy, and immunotherapies such as ICIs have been initiated. With ongoing technological advancements, OVs therapy is expected to achieve further breakthroughs and broaden its clinical potential in cancer treatment.

Clinical trials involving other emerging immune checkpoint-targeted immunotherapies in TNBC are summarized in Table 6. Currently, most investigational strategies are focused on ADCs targeting B7-H3 or B7-H4, several of which have entered phase I/II clinical evaluation in advanced solid tumors, including TNBC. In addition, B7-H3-targeted CAR-T therapy has also entered early clinical investigation for relapsed or refractory TNBC. Although current clinical evidence remains limited, these emerging immune checkpoint-targeted approaches highlight the continued expansion of next-generation immunotherapeutic strategies beyond conventional PD-1/PD-L1 blockade in TNBC.

Beyond currently available clinical trials, emerging immunotherapies such as cancer vaccines and oncolytic viruses have demonstrated substantial preclinical potential, either alone or in combination with anti-PD-1/PD-L1 therapies, by promoting antigen release, enhancing T cell activation, and converting immunologically “cold” tumors into more inflamed “hot” tumors.

Table 7 presents a comparative summary of immune checkpoint–based therapeutic strategies in TNBC.

## 5. Discussion

This review provides an overview of immune checkpoint pathways in the TNBC TME, summarizing the mechanisms of various immune checkpoint molecules and the current research and clinical applications of immune checkpoint-based immunotherapy in TNBC. Despite the clinical success of ICIs, their therapeutic efficacy remains limited to a subset of patients. Recently, several novel immunotherapeutic strategies, particularly the combination regimens integrating ICIs with chemotherapy, ADCs, tumor vaccines, and other targeted therapies, are being actively explored to overcome the current limitations of immunotherapy in TNBC.

The emergence of cancer immunotherapy has substantially reshaped the therapeutic landscape of TNBC, with an increasing number of immune checkpoints–based strategies entering clinical evaluation. Among these approaches, ICIs have demonstrated promising antitumor activity in TNBC [200,201]. In recent years, several predictive biomarkers have been investigated to improve patient stratification and optimize therapeutic responses to ICIs. Among them, PD-L1 expression currently represents the most clinically established biomarker for guiding immunotherapeutic strategies in TNBC. Patients with higher PD-L1 CPS generally derive greater clinical benefit from ICI-based chemotherapy regimens, whereas patients with low PD-L1 expression or acquired resistance to ICIs, particularly in mTNBC, often require intensified combination strategies to improve therapeutic efficacy [29,202].

Given the substantial molecular and immunological heterogeneity of TNBC, combination therapies involving ICIs with ADCs, PARP inhibitors, or other immunotherapeutic approaches have emerged as important strategies for overcoming immune resistance. For example, B7-H3- and B7-H4-targeted ADCs have shown promising translational potential in selected TNBC populations with high target expression. CAR-T cell therapy provides a strategy for redirecting tumor-specific T cell responses and overcoming immune resistance in patients with refractory TNBC. In addition, emerging therapeutic approaches such as cancer vaccines and OVs can enhance tumor immunogenicity, promote endogenous T cell activation, and potentially convert immunologically “cold” tumors into “hot” tumors, thereby improving the efficacy of ICIs when used in combination therapies. Furthermore, the development of personalized neoantigen-based immunotherapies has offered new opportunities for individualized treatment in TNBC [21,78].

Beyond the development of novel immunotherapeutic agents, innovative scaffold- and biomaterial-based platforms have emerged as promising strategies to enhance cancer immunotherapy. In particular, biomaterial scaffolds and nanotechnology-based delivery systems can modulate immune responses, enable localized delivery of therapeutic agents, and enhance immune–tumor interactions by reshaping the TME through activation of innate immune cells, including neutrophils, macrophages, and DCs [203]. For example, graphene oxide has been reported to promote DC maturation and cytokine secretion through the activation of multiple TLR signaling pathways [204]. In addition, lipid nanoparticles (LNPs), currently the most widely used delivery system for mRNA vaccines, have demonstrated intrinsic immunomodulatory properties. Studies have shown that LNPs can activate innate immune signaling pathways, including TLR4-associated pathways, promote DC maturation, enhance antigen presentation, and facilitate T cell activation, thereby increasing overall immunogenicity and strengthening antitumor immune responses [205,206]. Collectively, these scaffold- and biomaterial-based platforms may further enhance the efficacy of immunotherapy and represent a promising avenue for future TNBC treatment strategies.

Nevertheless, despite the encouraging progress achieved with immune checkpoint–based immunotherapy, several major challenges remain unresolved, including primary and acquired resistance, irAEs, and the lack of robust predictive biomarkers beyond PD-L1. Future studies should therefore focus on developing integrated biomarker-guided therapeutic strategies and rational combination regimens to further optimize the clinical efficacy and reduced toxicity, enabling more personalized therapeutic strategies for TNBC [62,145].

## 6. Conclusions and Future Directions

With the incorporation of ICIs combined with chemotherapy into the standard treatment paradigm for PD-L1-positive TNBC, immunotherapy has significantly advanced the clinical management of TNBC. However, several major limitations of current ICI-based therapies remain unresolved, including irAEs, primary and acquired resistance, and limited efficacy in specific patient populations. In particular, patients with PD-L1-negative metastatic TNBC and those previously refractory to anti-PD-1/PD-L1 therapies continue to exhibit poor responses to existing immunotherapeutic strategies. Moreover, some combination regimens involving ICIs may be associated with substantial immune-related toxicities, highlighting the need for safer and more effective therapeutic alternatives [37,207].

Emerging immune checkpoint targets, including LAG-3, TIM-3, TIGIT, B7-H3, and B7-H4, as well as novel therapeutic modalities such as BsAbs, ADCs and other therapies, have expanded the immunotherapeutic landscape of TNBC. Consequently, immunotherapy for TNBC is evolving from single-agent PD-1/PD-L1 inhibition toward multi-target and combination-based strategies. However, most of these strategies remain in early-phase clinical development and still lack large-scale phase III clinical validation. In addition, combination strategies integrating ICIs with ADCs, tumor vaccines, and OVs represent promising future directions, particularly for immunologically “cold” TNBC.

Despite the clinical utility of PD-L1 as the established biomarker for selecting TNBC patients for ICI therapy, other predictive markers, including TILs, TMB, and HLA-related features, remain limited due to the substantial molecular and immunological heterogeneity of TNBC. Furthermore, the lack of preclinical models that faithfully recapitulate this immune heterogeneity hampers biomarker development. Moving forward, research should prioritize the creation of integrated biomarker models to optimize patient stratification, overcome therapeutic resistance, and establish rational, personalized immunotherapeutic strategies. Adaptive platform trial designs, incorporating biomarker-guided combination strategies based on liquid biopsy and tumor immune profiling, should be further explored to refine and advance personalized immunotherapy approaches for TNBC [82,208].

## Figures and Tables

**Table 1:** Summary of clinical trials of anti-PD-1/PD-L1 monoclonal antibodies pembrolizumab and atezolizumab in TNBC.

Register ID	Phase	Status	Drugs	Drug Targets	Study Population	Major Outcomes
KEYNOTE-012 (NCT01848834)	Phase I	Completed	Pembrolizumab	PD-1	297 patients with advanced TNBC	ORR: 18.5%; mOS: 11.2 months; mPFS: 1.9 months
KEYNOTE-086 (NCT02447003)	Phase II	Completed	Pembrolizumab	PD-1	Part 1: 170 TNBC patients who received no prior systemic treatment	ORR: 5.3%; mOS: 9.0 months; mPFS: 2.0 months
					Part 2: 84 PD-L1-positive mTNBC patients who received no prior systemic treatment	ORR: 21.4%, mOS: 18.0 months; mPFS: 2.1 months
KEYNOTE-522 (NCT03036488)	Phase III	Ongoing	Pembrolizumab + chemotherapy vs. placebo + chemotherapy	PD-1	1174 patients untreated high-risk early-stage TNBC	OS at 60 months: 86.8% (Pembrolizumab) vs. 81.1% (placebo)
KEYNOTE-355 (NCT02819518)	Phase III	Completed	Pembrolizumab plus chemotherapy vs. placebo plus chemotherapy	PD-1	882 patients with previously untreated locally recurrent inoperable or metastatic TNBC	CPS-10 subgroup: mOS: 23.0 months (Pembrolizumab–chemotherapy group) 16.1 months (placebo–chemotherapy group)
KEYNOTE-119 (NCT02555657)	Phase III	Completed	Single agent Pembrolizumab vs. single agent chemotherapy	PD-1	622 patients with metastatic TNBC	CPS-10 subgroup mOS: 12.7 months (Pembrolizumab group) vs. 11.6 months (the chemotherapy group)
NCT01375842	Phase I	Completed	Atezolizumab	PD-L1	116 patients with metastatic TNBC	In first-line patients: mOS: 17.6 months; ORR: 24%
IMpassion031 (NCT03197935)	Phase III	Completed	Atezolizumab vs. placebo in combination with chemotherapy	PD-L1	333 patients with early-stage TNBC	All participants: pCR rate: 58% (Atezolizumab) vs. 41% (placebo); PD-L1-positive participants: pCR rate: 69% (Atezolizumab) vs. 49% (placebo)
IMpassion130 (NCT02425891)	Phase III	Completed	Atezolizumab in combination with nab-paclitaxel compared with placebo with nab-paclitaxel	PD-L1	902 patients diagnosed with previously untreated metastatic TNBC	mPFS: 7.2 months (Atezolizumab plus nab-paclitaxel) vs. 5.5 months (placebo plus nab-paclitaxel); mOS: 21.3 months (Atezolizumab plus nab-paclitaxel) vs. 17.6 months (placebo plus nab-paclitaxel)
IMpassion131 (NCT03125902)	Phase III	Completed	Atezolizumab and paclitaxel vs. placebo and paclitaxel	PD-L1	653 patients with previously untreated locally advanced or metastatic TNBC	mPFS: 6.0 months (Atezolizumab-paclitaxel) vs. 5.7 months (placebo-paclitaxel) mOS: 22.1 months (Atezolizumab-paclitaxel) vs. 28.3 months (placebo-paclitaxel)
IMpassion132 (NCT03371017)	Phase III	Completed	Atezolizumab vs. placebo in combination with chemotherapy	PD-L1	595 TNBC patients with early relapsing recurrent	ORR: 40% (Atezolizumab) vs. 28% (placebo); mOS: 12.1 months (Atezolizumab) vs. 11.2 months (placebo)
NCT03289819	Phase II	Completed	Pembrolizumab/nab-paclitaxel followed by pembrolizumab/epirubicin/cyclophosphamide	PD-1	53 TNBC patients	pCR rate: 71.8%
NCT02883062	Phase II	Active, not recruiting	Carboplatin and paclitaxel with or without Atezolizumab	PD-L1	67 patients with newly diagnosed, stage II-III TNBC	NA
NCT03281954	Phase III	Active, not recruiting	Neoadjuvant chemotherapy with Atezolizumab or placebo	PD-L1	1550 TNBC patients followed after surgery by Atezolizumab or placebo	NA

NA—Not Applicable.

**Table 2:** Summary of clinical trials of other anti-PD-1/PD-L1 monoclonal antibodies in TNBC.

Register ID	Phase	Status	Drugs	Drug Targets	Study Population	Major Outcomes
NCT02628132	Phase I; Phase II	Completed	Durvalumab in combination with paclitaxel	PD-L1	25 TNBC patients	NA
GeparNuevo (NCT02685059)	Phase II	Completed	Durvalumab vs. placebo in combination with Taxane-anthracycline	PD-L1	174 TNBC patients	pCR rate: 53.4% (Durvalumab) vs. 44.2% (placebo)
NCT03487666	Phase II	Completed	Nivolumab or capecitabine or combination therapy	PD-1	45 TNBC patients with residual disease	NA
NCT02393794	Phase I; Phase II	Active, not recruiting	Cisplatin plus romidepsin and Nivolumab	PD-1	51 patients with locally recurrent or metastatic TNBC	NA
NCT05888831	Phase I; Phase II	Active, not recruiting	BMS-986449 with and without Nivolumab	PD-1	100 patients with advanced solid tumors (Part 1C is TNBC patients.)	NA
NCT03330405	Phase I; Phase II	Terminated	Avelumab plus Talazoparib	PD-L1	223 patients with locally advanced or metastatic solid tumors	ORR was 18.2%; mDOR: 11.1 months in TNBC patients
NCT03971409	Phase II	Recruiting	Avelumab with Binimetinib, Sacituzumab govitecan, or liposomal doxorubicin	PD-L1	150 patients with stage IV or unresectable, recurrent TNBC	NA
NCT02926196	Phase III	Completed	Avelumab	PD-L1	474 high-risk TNBC Patients	3-year DFS: patients in the intention-to-treat population: 68.3% (Avelumab) vs. 63.2% (control arm). 3-year OS: 84.8% (Avelumab) vs. 76.3% (control arm)

NA—Not Applicable.

**Table 3:** Summary of clinical trials of novel combination therapies anti-PD-1/PD-L1 in TNBC.

Register ID	Phase	Status	Drugs	Drug Targets	Study Population	Major Outcomes
NCT04468061	Phase II	Recruiting	Pembrolizumab with or without sacituzumab govitecan	PD-1	110 patients with metastatic TNBC	NA
NCT04434040	Phase II	Recruiting	Atezolizumab + sacituzumab govitecan	PD-L1	40 TNBC patients	NA
NCT06393374	Phase III	Recruiting	Sacituzumab tirumotecan (MK-2870) in combination with pembrolizumab compared to treatment of physician’s choice	PD-1	1530 TNBC patients who did not achieve pCR	NA
NCT06841354	Phase III	Recruiting	Sacituzumab tirumotecan in combination with pembrolizumab	PD-1	1000 participants with previously untreated locally recurrent unresectable or metastatic TNBC expressing PD-L1 at CPS less than 10	NA
NCT04504669	Phase III	Recruiting	Dato-DXd with or without durvalumab	PD-L1	625 patients with PD-L1 positive locally recurrent inoperable or metastatic TNBC	NA
NCT06112379	Phase III	Active, not recruiting	Dato-DXd in combination with durvalumab	PD-L1	1902 patients with triple-negative or hormone receptor-low/HER2-negative breast cancer	NA
NCT05629585	Phase III	Active, not recruiting	Dato-DXd with or without durvalumab	PD-L1	1174 in patients with stage I-III TNBC without pCR following neoadjuvant therapy	NA
NCT04504669	Phase I	Completed	AZD8701 alone and in combination with durvalumab	PD-L1	60 patients with advanced solid tumors	NA
NCT03289962	Phase I	Completed	Autogene cevumeran (RO7198457) as a single agent and in combination with atezolizumab	PD-L1	272 patients with locally advanced or metastatic tumors	NA
NCT03761914	Phase I; Phase II	Completed	Galinpepimut-S in combination with pembrolizumab	PD-1	26 patients with selected advanced cancers	NA
NCT05269381	Phase I; Phase II	Recruiting	Personalized neoantigen peptide-based vaccine in combination with pembrolizumab	PD-1	36 patients with advanced solid tumors	NA
NCT04176848	Phase II	Active, not recruiting	CFI-400945 and durvalumab	PD-L1	15 in patients with advanced TNBC	NA
KEYNOTE-162 (NCT02657889)	Phase I; Phase II	Completed	Niraparib in combination with pembrolizumab	PD-1	122 TNBC patients or ovarian cancer	ORR: 21%; DCR: 49%
NCT02849496	Phase II	Active, not recruiting	Olaparib either alone or in combination with atezolizumab	PD-L1	81 patients in BRCA mutant non-HER2-positive breast cancer	In TNBC subgroup (*n* = 23), PFS: 7.0 months (Olaparib) and 7.67 months (Olaparib + Atezolizumab). mOS: 26.5 months (Olaparib) and 22.4 months (Olaparib + Atezolizumab).
NCT05064280	Phase II	Recruiting	Pembrolizumab in combination with lenvatinib	PD-1	104 TNBC patients NSCLC, and other tumor types and brain metastases	NA
NCT05203445	Phase II	Active, not recruiting	Olaparib and pembrolizumab	PD-1	23 TNBC patients or hormone receptor-positive HER2-negative breast cancer	NA
NCT04191135	Phase II	Active, not recruiting	Olaparib plus pembrolizumab vs. chemotherapy plus pembrolizumab	PD-1	462 TNBC patients	NA

NA—Not Applicable.

**Table 4:** Summary of clinical trials involving monoclonal antibodies targeting other immune checkpoints in TNBC.

Register ID	Phase	Status	Drugs	Drug Targets	Study Population
NCT03818685	Phase II	Active, not recruiting	Radiotherapy + Nivolumab (anti-PD-1) + Ipilimumab (anti-CTLA-4) vs. radiotherapy + capecitabine	PD-1; CTLA-4	95 TNBC patients with residual disease
NCT06342037	Phase II	Recruiting	Tiragolumab (anti-TIGIT) with atezolizumab (anti-PD-L1) and/or ipilimumab (anti-CTLA-4)	TIGIT with PD-L1 and CTLA-4	60 patients with advanced TNBC
NCT03815890	Phase II	Recruiting	Nivolumab (anti-PD-1) + ipilimumab	PD-L1 and CTLA-4	80 patients with early-stage TNBC
NCT03546686	Phase II	Recruiting	Immune checkpoint inhibition (pembrolizumab; ipilimumab; nivolumab)	PD-1 and CTLA-4	80 patients with residual triple-negative resectable breast cancer after taxane-based neoadjuvant chemotherapy
NCT02527434	Phase II	Completed	Tremelimumab	CTLA-4	64 patients with advanced solid tumors
NCT03606967	Phase II	Recruiting	Durvalumab (anti-PD-L1) and tremelimumab (anti-CTLA-4) and chemotherapy in addition to individualized vaccine	PD-L1 and CTLA-4	70 patients with metastatic TNBC
NCT03518606	Phase I; Phase II	Completed	Durvalumab (anti-PD-L1) + tremelimumab (anti-CTLA-4) + metronomic vinorelbine	PD-L1 and CTLA-4	126 patients with advanced solid tumors (19 TNBC patients)
NCT02460224	Phase I; Phase II	Completed	Ieramilimab (LAG525)	LAG-3	490 patients with advanced malignancies
NCT03499899	Phase II	Completed	LAG525 (anti-LAG-3) in combination with spartalizumab (anti-PD-1), or with spartalizumab and carboplatin, or with carboplatin	LAG-3	88 patients with advanced TNBC
NCT03652077	Phase I	Completed	INCAGN02390	TIM-3	40 patients with select advanced malignancies
NCT04584112	Phase I	Completed	Tiragolumab in combination with atezolizumab and chemotherapy	TIGIT and PD-L1	83 TNBC Patients
NCT06175390	Phase II	Recruiting	Tiragolumab, atezolizumab and chemotherapy	TIGIT and PD-L1	130 TNBC patients (cohort A in early TNBC patients and cohort B in late in metastatic TNBC patients)
NCT07134556	Phase II	Not yet recruiting	Zimberelimab (anti-PD-1), domvanalimab (anti-TIGIT) and sacituzumab govitecan	PD-1 and TIGIT	25 patients with PD-L1 positive advanced or metastatic TNBC
NCT07189871	Phase I; Phase II	Not yet recruiting	177Lu-BetaBart	B7-H3	61 patients with relapsed/refractory, locally advanced inoperable, or metastatic solid tumors

**Table 5:** Summary of clinical trials involving bispecific antibodies targeting immune checkpoints in TNBC.

Register ID	Phase	Status	Drugs	Drug Targets	Study Population
NCT03872791	Phase Ib/II	Completed	KN046 alone or in combination with nab-paclitaxel	PD-1/CTLA-4	52 patients with locally advanced unresectable or metastatic TNBC.
NCT04606472	Phase I	Recruiting	SI-B003	PD-1/CTLA-4	159 patients with advanced solid tumors
NCT03219268.	Phase I	Completed	MGD013	PD-1/LAG-3	277 patients with unresectable or metastatic neoplasms
NCT07173751	Phase III	Not yet recruiting	BNT327 (Pumitamig)	PD-L1/VEGF-A	558 patients with previously untreated locally recurrent inoperable or metastatic TNBC
NCT03849469	Phase I	Completed	XmAb22841 monotherapy and in combination with pembrolizumab (anti-PD-1)	CTLA-4/LAG-3	78 patients with selected advanced solid tumors
NCT05620134	Phase I/II	Active, not recruiting	JK08	CTLA-4/IL-15	263 patients with unresectable locally advanced or metastatic cancer
NCT07158918	Phase I/II	Recruiting	ABL103 plus pembrolizumab (anti-PD-1), with or without taxane,	B7-H4/CD137 (4-1BB)	65 patients with advanced or metastatic solid tumors
NCT05852691	Phase II	Active, not recruiting	Tobemstomig + nab-paclitaxel compared with pembrolizumab + nab-paclitaxel	PD-1/LAG-3	83 participants with previously untreated, PD-L1-positive, locally advanced unresectable or metastatic TNBC

**Table 6:** Summary of clinical trials of other immunotherapies targeting immune checkpoints in TNBC.

Register ID	Phase	Status	Drugs	Drug Targets	Types	Study Population
NCT06554795	Phase I/IIa	Recruiting	DB-1419	B7-H3 and PD-L1	ADC	360 patients with advanced/metastatic solid tumors
NCT03729596	Phase I; Phase II	Terminated	MGC018 and MGA012 (PD-1 inhibitor)	B7-H3 and PD-1	ADC	143 patients with solid tumors (patients with locally advanced or metastatic TNBC who have progressed after at least one systemic therapy received 3.0 mg/kg intravenously every 3 weeks)
NCT05123482	Phase I/IIa	Recruiting	AZD8205	B7-H4	ADC	370 patients with advanced solid Tumors
NCT05377996	Phase I	Recruiting	XMT-1660	B7-H4	ADC	319 patients with solid tumors
NCT06774963	Phase I	Recruiting	LNCB74	B7-H4	ADC	145 patients with advanced solid tumors
NCT06233942	Phase I	Recruiting	BG-C9074 alone and in combination with Tislelizumab	B7-H4	ADC	227 patients with advanced solid tumors
NCT06347068	Phase I	Recruiting	iC9-CAR	B7-H3	CAR-T	42 relapsed/refractory TNBC patients

**Table 7:** Comparative summary of immune checkpoint–based therapeutic strategies in TNBC.

Target	Strategy	Clinical Stage	Limitations and Challenges
PD-1	ICI	Approved (Pembrolizumab), others are in Phase I–III trials	ICIs are associated with irAEs and limited predictive biomarkers, resistance mechanisms remain incompletely understood. Emerging therapeutic strategies are in the early stages of development.
	BsAb	Phase I/II	
	ADC	Phase I/II	
	Tumor vaccine	Preclinical (PD-1 B cell epitope peptide)	
	OV	Preclinical (PD-1v)	
PD-L1	ICI	Approved (Atezolizumab); others are in Phase I–III trials	
	BsAb	Phase III	
	ADC	Phase I/II	
	Tumor vaccine	Preclinical	
	OV	Preclinical	
CTLA-4	ICI	Phase I/II	ICIs has limited efficacy; irAEs, combination optimization required
	BsAb	Phase I/II	
LAG-3	ICI	Phase I/II	Limited TNBC-specific clinical data; early-stage clinical development; lack of validated predictive biomarkers
	BsAb	Phase I/II	
TIM-3	ICI	Phase I	
TIGIT	ICI	Phase I/II	
B7-H3	ICI	Phase I/II	Limited clinical validation; early-stage development, potential treatment resistance; cumulative toxicity.
	BsAb	Preclinical	
	ADC	Phase I/II	
	CAR-T	Preclinical → Phase I/II	
B7-H4	BsAb	Preclinical → Phase I/II	
	ADC	Phase I	

## Data Availability

No new data were created or analyzed in this study. Data sharing is not applicable to this article.

## Abbreviations

The following abbreviations are used in this manuscript:

ADC	Antibody-Drug Conjugates
ADCC	Antibody-Dependent Cell-mediated Cytotoxicity
α-LA	α-Lactalbumin
APC	Antigen-Presenting Cell
BsAb	Bispecific Antibody
CAR	Chimeric Antigen Receptor
CCL	Chemoattractant Cytokine Ligand
CD	Cluster of Differentiation
CPS	Combined Positive Score
CTLA-4	Cytotoxic T Lymphocyte-associated Antigen-4
DALYs	Disability-Adjusted Life-Years
DAMP	Damage associated molecular patterns
DAR	Drug-to-Antibody Ratio
DC	Dendritic cell
DCR	Disease Control Rate
ER	Estrogen Receptor
FoxP3	Forkhead Box P3
GM-CSF	Granulocyte-Macrophage Colony Stimulating Factor
HER2	Human Epidermal growth factor Receptor 2
HLA	Human Leukocyte Antigen
ICB	Immune Checkpoint Blockade
ICD	Immunogenic cell death
ICI	Immune checkpoint inhibitor
IFN-γ	Interferon gamma
IL	Interleukin
irAE	immune-related Adverse Event
LAG-3	Lymphocyte Activation Gene-3
LNP	lipid nanoparticle
MHC	Major Histocompatibility Complex
MSC	Mesenchymal Stem Cells
NK cell	Natural killer cell
ORR	Overall Response Rate
OS	Overall Survival
OV	Oncolytic Virus
PARP	Poly ADP-Ribose Polymerase
PBMC	Peripheral Blood Monoculear Cell
pCR	Pathological Complete Response
PD-1	Programmed death 1
PD-L1	Programmed cell death-Ligand 1
PDX	Patient-Derived Xenograft
PFS	Progression-Free Survival
PR	Progesterone Receptor
PtdSer	Phosphatidylserine
RCB	Residual Cancer Burden
SG	Sacituzumab govitecan
STAT	Signal Transducer and Activator of Transcription
TAA	Tumor Associated Antigen
TAM	Tumor-Associated Macrophages
TCR	T cell receptor
Th cell	Helper T cell
TIGIT	T cell immunoreceptor with immunoglobulin and ITIM domain
TIL	Tumor Infiltrating Lymphocytes
TIM-3	T cell immunoglobulin and mucin domain-containing protein 3
TMB	tumor mutation burden
TME	Tumor Microenvironment
TLR	Toll-like Receptors
TNBC	Triple-Negative Breast Cancer
TNF	Tumor Necrosis Factor
Treg cell	Regulatory T cell
Trop-2	Trophoblast cell surface antigen 2
TSA	Tumor Specific Antigen
T-VEC	Talimogene laherparepvec
ZAP70	Zeta Chain of T Cell Receptor Associated Protein Kinase 70
